# Crystal structure of azilsartan methyl ester ethyl acetate hemisolvate

**DOI:** 10.1107/S2056989014028023

**Published:** 2015-01-03

**Authors:** Zhengyi Li, Rong Liu, Meilan Zhu, Liang Chen, Xiaoqiang Sun

**Affiliations:** a Jiangsu Key Laboratory of Advanced Catalytic Materials and Technology, School of Petrochemical Engineering, Changzhou University, Changzhou 213164, Jiangsu, People’s Republic of China; b Changzhou Siyao Pharmaceuticals Co., Ltd, Changzhou 213000, Jiangsu, People’s Republic of China

**Keywords:** crystal structure, azilsartan, azilsartan methyl ester, anti­hypertension

## Abstract

The title compound, C_26_H_22_N_4_O_5_ (systematic name: methyl 2-eth­oxy-1-{4-[2-(5-oxo-4,5-di­hydro-1,2,4-oxa­diazol-3-yl)phenyl]benz­yl}-1*H*-1,3-benzo­diazole-7-carboxyl­ate ethyl acetate hemisolvate), was obtained *via* cyclization of methyl (*Z*)-2-eth­oxy-1-{(2′-(*N*′-hy­droxy­carbamimido­yl)-[1,1′-biphen­yl]-4-yl)meth­yl}-1*H*-benzo[*d*]imidazole-7-carboxyl­ate with diphen­yl carbonate. There are two independent mol­ecules (*A* and *B*) with different conformations and an ethyl acetate solvent mol­ecule in the asymmetric unit. In mol­ecule *A*, the dihedral angle between the benzene ring and its attached oxa­diazole ring is 59.36 (17); the dihedral angle between the benzene rings is 43.89 (15) and that between the benzene ring and its attached imidazole ring system is 80.06 (11)°. The corres­ponding dihedral angles in mol­ecule *B* are 58.45 (18), 50.73 (16) and 85.37 (10)°, respectively. The C—O—C—C_m_ (m = meth­yl) torsion angles for the eth­oxy side chains attached to the imidazole rings in mol­ecules *A* and *B* are 93.9 (3) and −174.6 (3)°, respectively. In the crystal, the components are linked by N—H⋯N and C—H⋯O hydrogen bonds, generating a three-dimensional network. Aromatic π–π stacking inter­actions [shortest centroid–centroid separation = 3.536 (3)Å] are also observed.

## Related literature   

For general background to azilsartan, an angiotensin II type 1 (AT1) receptor blocker (ARB) having a perfect anti­hypertensive effect, see: Michel *et al.* (2013 ▸); Weltman *et al.* (2012 ▸); Ojima *et al.* (2011 ▸). For the synthesis of azilsartan methyl ester, the key synthetic inter­mediate of azilsartan, see: Kohara *et al.* (1996 ▸); Rádl *et al.* (2013 ▸).
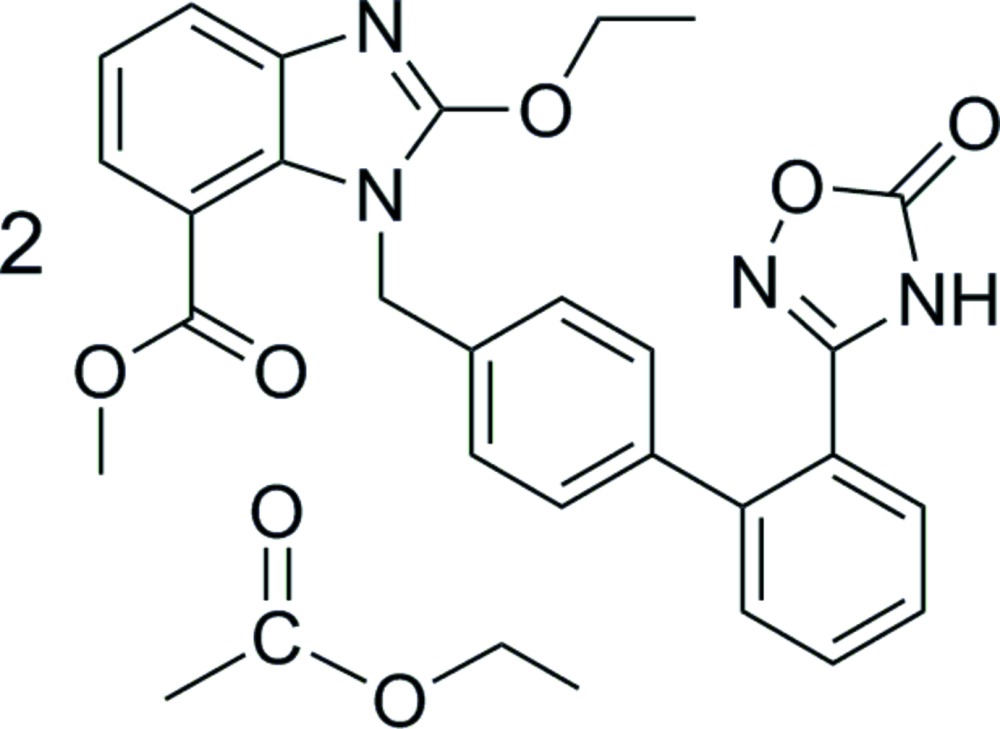


## Experimental   

### Crystal data  

2C_26_H_22_N_4_O_5_·C_4_H_8_O_2_
*M* *_r_* = 1029.06
Triclinic, 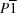

*a* = 13.662 (5) Å
*b* = 14.928 (6) Å
*c* = 15.356 (10) Å
α = 95.459 (11)°
β = 106.226 (11)°
γ = 116.524 (8)°
*V* = 2601 (2) Å^3^
*Z* = 2
Mo *K*α radiation
μ = 0.09 mm^−1^
*T* = 293 K
0.25 × 0.20 × 0.18 mm

### Data collection  

Bruker APEXII CCD diffractometer
Absorption correction: multi-scan (*SADABS*; Bruker, 2009 ▸) *T*_min_ = 0.977, *T*_max_ = 0.983
14182 measured reflections
9024 independent reflections
6365 reflections with *I* > 2σ(*I*)
*R*_int_ = 0.030

### Refinement  

*R*[*F* ^2^ > 2σ(*F* ^2^)] = 0.064
*wR*(*F* ^2^) = 0.221
*S* = 1.01
9024 reflections
699 parameters
H atoms treated by a mixture of independent and constrained refinement
Δρ_max_ = 0.55 e Å^−3^
Δρ_min_ = −0.28 e Å^−3^

### 

Data collection: *APEX2* (Bruker, 2009 ▸); cell refinement: *SAINT* (Bruker, 2009 ▸); data reduction: *SAINT*; program(s) used to solve structure: *SHELXTL* (Sheldrick, 2008 ▸); program(s) used to refine structure: *SHELXTL*; molecular graphics: *SHELXTL*; software used to prepare material for publication: *SHELXTL*.

## Supplementary Material

Crystal structure: contains datablock(s) I, global. DOI: 10.1107/S2056989014028023/hb7346sup1.cif

Structure factors: contains datablock(s) I. DOI: 10.1107/S2056989014028023/hb7346Isup2.hkl

Supporting information file. DOI: 10.1107/S2056989014028023/hb7346Isup3.cml

**Fig. 1.**
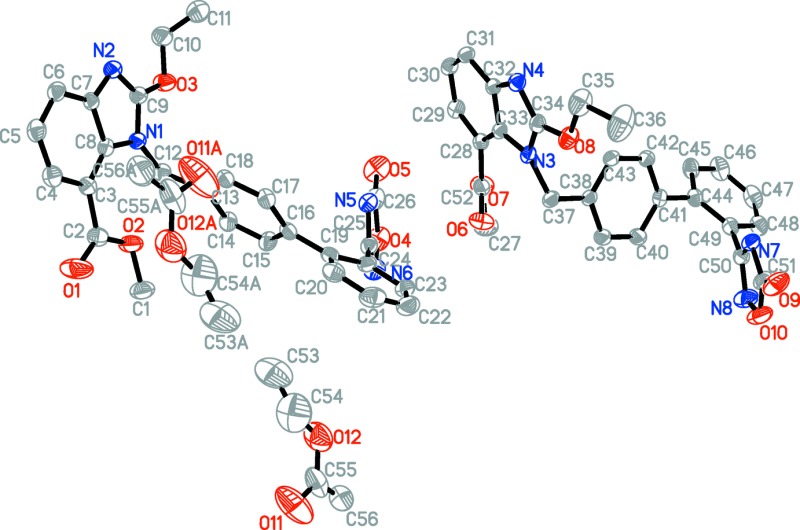
The molecular structure of the title compound. Displacement ellipsoids are drawn at the 30% probability level. All H atoms have been omitted for clarity.

. DOI: 10.1107/S2056989014028023/hb7346fig1.tif
The mol­ecular structure of the title compound. Displacement ellipsoids are drawn at the 30% probability level. All H atoms have been omitted for clarity.

**Fig. 2.**
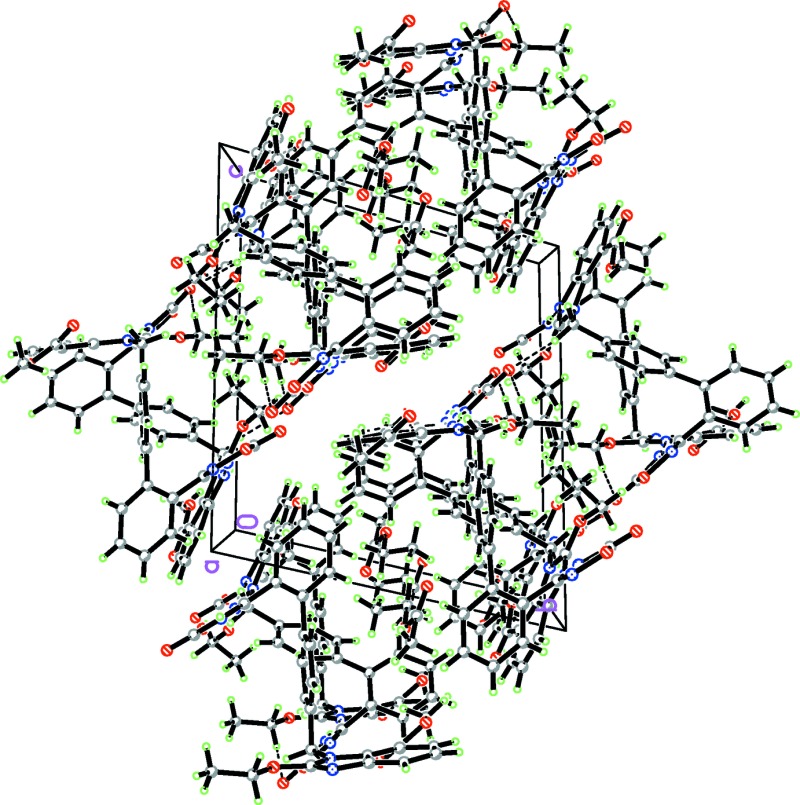
Crystal packing of (I). Hydrogen bonds are shown as dashed lines.

. DOI: 10.1107/S2056989014028023/hb7346fig2.tif
Crystal packing of (I). Hydrogen bonds are shown as dashed lines.

CCDC reference: 934880

Additional supporting information:  crystallographic information; 3D view; checkCIF report

## Figures and Tables

**Table 1:** Hydrogen-bond geometry (, )

*D*H*A*	*D*H	H*A*	*D* *A*	*D*H*A*
N5H5*A*N4^i^	1.01	1.85	2.862(3)	176
N7H7N2^i^	0.90	2.01	2.905(3)	173
C12H12*A*O5^ii^	0.97	2.58	3.421(4)	145
C30H30O9^iii^	0.93	2.58	3.484(3)	163

Symmetry codes: (i) 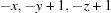; (ii) 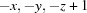; (iii) 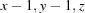.

## supplementary crystallographic information

### S1. Comment

Azilsartan (TAK-536) as an angiotensin II type 1 (AT1) receptor blocker (ARB) has perfect antihypertensive effect (Ojima *et al.*, 2011; Michel *et al.*, 2013; Weltman *et al.*, 2012). Azilsartan methyl ester, as the key synthetic intermediate of azilsartan has being paid widely attention. Although the synthesis of azilsartan methyl ester has been descripted in many literatures (Kohara *et al.*, 1996; Rádl *et al.*, 2013), there was no particular work on the structural characterization involving crystal structure. We herein present the crystal structure of methyl 2-ethoxy-1-((2'-(5-oxo-4,5-dihydro-1,2,4-oxadiazol-3-yl) -[1,1'-biphenyl]-4-yl)methyl)-1*H*-benzo[*d*]imidazole-7- carboxylate (I).

In the molecular structure (Fig. 1), there are two independent title molecules with different conformations and an ethyl acetate solvent molecule to form the minimum repeat unit of the crystal. The dihedral angles between the two phenyl rings (C38–C43 with C44–C49; C13–C18 with C19–C24) of phenylbenzenes are 50.7 (2)° and 43.9 (1)°, respectively. The new constructed 5-oxo-1,2,4-oxadiazole heterocycle (N5–C26–O4–N6–C25 or N7–C51–O10–N8–C50) adopts a planer structure. In the packing structure (Fig. 2), the two different title compound molecules formed a dimer through two intermolecular N–H···N hydrogen bond (N5–H5A···N4 and N7–H7···N2) between the imidazole rings and 5-oxo-1,2,4-oxadiazole rings. Then intermolecular π–π stacking (*C*g1···*C*g2^i^, 3.62 (2) Å, symmetry codes: (i), -*x*, 1 - *y*, 1 - *z. C*g1 and *C*g2 are the centroids of the N3–C33–C32–N4–C34 and C28–C33 rings, respectively) between the two benzo[*d*]imidazole rings and C–H···O weak interactions (C12–H12A···O5 and C30–H30···O9) link the adjacent dimers.

### S2. Experimental

A mixture of methyl (*Z*)-2-ethoxy-1-((2'-(*N*'-hydroxycarbamimidoyl) -[1,1'-biphenyl]-4-yl)methyl)-1*H*-benzo[*d*]imidazole-7-carboxylate (6 g, 13.5 mmol), diphenyl carbonate (4.34 g, 20.25 mmol) and K_2_CO_3_ (2.8 g, 20.25 mmol) in DMSO (150 ml) was stirred at room temperature for 5 h. After the reaction, the mixture was poured into water (400 ml) and the insoluble portion was filtered off. The aqueous solution was acidified with 1 N HCl to PH = 3–4, and the formed precipitate was filtered off, washed with sodium carbonate solution, and dried *in vacuo* to afford a white solid (5.28 g, 83% yield, m.p. 468–470 K). Colourless blocks were obtained by slow evaporation of an ethyl acetate solution at room temperature.

### S3. Refinement

All the H bonded to C atoms were placed in geometrically idealized positions and constrained to ride on their parent atoms, with C—H distances of 0.93–0.97 Å, and with *U*_iso_(H) = 1.2–1.5*U*_eq_(C). All the H on N atoms were located in a differences Fourier map and refined isotropically.

### Crystal data

**Table:**

C_26_H_22_N_4_O_5_·0.5C_4_H_8_O_2_	*Z* = 4
*M**_r_* = 514.53	*F*(000) = 1080
Triclinic, *P*1	*D*_x_ = 1.314 Mg m^−^^3^
Hall symbol: -P 1	Mo *K*α radiation, λ = 0.71073 Å
*a* = 13.662 (5) Å	Cell parameters from 5677 reflections
*b* = 14.928 (6) Å	θ = 2.4–28.3°
*c* = 15.356 (10) Å	µ = 0.09 mm^−^^1^
α = 95.459 (11)°	*T* = 293 K
β = 106.226 (11)°	BLOCK, colorless
γ = 116.524 (8)°	0.25 × 0.20 × 0.18 mm
*V* = 2601 (2) Å^3^	

### Data collection

**Table:**

Bruker APEXII CCD diffractometer	9024 independent reflections
Radiation source: fine-focus sealed tube	6365 reflections with *I* > 2σ(*I*)
Graphite monochromator	*R*_int_ = 0.030
phi and ω scans	θ_max_ = 25.0°, θ_min_ = 1.4°
Absorption correction: multi-scan (*SADABS*; Bruker, 2009)	*h* = −16→16
*T*_min_ = 0.977, *T*_max_ = 0.983	*k* = −12→17
14182 measured reflections	*l* = −16→18

### Refinement

**Table:**

Refinement on *F*^2^	Primary atom site location: structure-invariant direct methods
Least-squares matrix: full	Secondary atom site location: difference Fourier map
*R*[*F*^2^ > 2σ(*F*^2^)] = 0.064	Hydrogen site location: inferred from neighbouring sites
*wR*(*F*^2^) = 0.221	H atoms treated by a mixture of independent and constrained refinement
*S* = 1.01	*w* = 1/[σ^2^(*F*_o_^2^) + (0.1569*P*)^2^ + 0.180*P*] where *P* = (*F*_o_^2^ + 2*F*_c_^2^)/3
9024 reflections	(Δ/σ)_max_ = 0.001
699 parameters	Δρ_max_ = 0.55 e Å^−^^3^
0 restraints	Δρ_min_ = −0.28 e Å^−^^3^

### Special details

**Table:**

Geometry. All e.s.d.'s (except the e.s.d. in the dihedral angle between two l.s. planes) are estimated using the full covariance matrix. The cell e.s.d.'s are taken into account individually in the estimation of e.s.d.'s in distances, angles and torsion angles; correlations between e.s.d.'s in cell parameters are only used when they are defined by crystal symmetry. An approximate (isotropic) treatment of cell e.s.d.'s is used for estimating e.s.d.'s involving l.s. planes.
Refinement. Refinement of *F*^2^ against ALL reflections. The weighted *R*-factor *wR* and goodness of fit *S* are based on *F*^2^, conventional *R*-factors *R* are based on *F*, with *F* set to zero for negative *F*^2^. The threshold expression of *F*^2^ > σ(*F*^2^) is used only for calculating *R*-factors(gt) *etc*. and is not relevant to the choice of reflections for refinement. *R*-factors based on *F*^2^ are statistically about twice as large as those based on *F*, and *R*- factors based on ALL data will be even larger.

### Fractional atomic coordinates and isotropic or equivalent isotropic displacement parameters (Å^2^)

**Table:**

	*x*	*y*	*z*	*U*_iso_*/*U*_eq_	
N4	−0.00150 (17)	0.67033 (16)	0.44581 (13)	0.0415 (5)	
C32	−0.0207 (2)	0.57119 (19)	0.41187 (14)	0.0369 (5)	
C33	0.08424 (19)	0.57566 (18)	0.41020 (14)	0.0364 (5)	
N3	0.16847 (17)	0.68218 (15)	0.44370 (13)	0.0385 (5)	
O8	0.17051 (17)	0.83549 (14)	0.48946 (14)	0.0570 (5)	
C28	0.0874 (2)	0.48615 (19)	0.37778 (15)	0.0395 (6)	
C31	−0.1238 (2)	0.4749 (2)	0.38319 (16)	0.0435 (6)	
H31	−0.1924	0.4704	0.3874	0.052*	
N7	0.49747 (19)	1.00469 (17)	0.19708 (15)	0.0473 (6)	
O7	0.18151 (18)	0.41080 (18)	0.31586 (14)	0.0672 (6)	
C42	0.2215 (2)	0.7413 (2)	0.18920 (16)	0.0443 (6)	
H42	0.1596	0.7347	0.1391	0.053*	
C37	0.2891 (2)	0.7362 (2)	0.44727 (16)	0.0428 (6)	
H37A	0.3301	0.7020	0.4770	0.051*	
H37B	0.3280	0.8065	0.4859	0.051*	
C43	0.2089 (2)	0.7316 (2)	0.27565 (17)	0.0438 (6)	
H43	0.1387	0.7191	0.2824	0.053*	
C48	0.4438 (3)	0.8462 (3)	−0.0169 (2)	0.0594 (8)	
H48	0.5087	0.8993	−0.0237	0.071*	
N8	0.6348 (2)	0.9740 (3)	0.1776 (2)	0.0751 (8)	
C38	0.29870 (19)	0.74027 (18)	0.35088 (15)	0.0376 (5)	
C51	0.6010 (2)	1.0861 (3)	0.2611 (2)	0.0578 (8)	
C30	−0.1222 (2)	0.3874 (2)	0.34885 (16)	0.0452 (6)	
H30	−0.1916	0.3241	0.3266	0.054*	
C47	0.3581 (3)	0.7671 (3)	−0.0945 (2)	0.0671 (9)	
H47	0.3653	0.7662	−0.1530	0.081*	
C41	0.3261 (2)	0.76074 (19)	0.17754 (16)	0.0420 (6)	
O9	0.61870 (19)	1.16176 (18)	0.31497 (16)	0.0799 (8)	
C34	0.1098 (2)	0.73142 (19)	0.46168 (16)	0.0410 (6)	
O6	0.29152 (18)	0.54126 (19)	0.44628 (16)	0.0711 (6)	
C49	0.4348 (2)	0.8478 (2)	0.07188 (18)	0.0464 (6)	
C52	0.1984 (2)	0.4840 (2)	0.38488 (18)	0.0459 (6)	
C50	0.5237 (2)	0.9410 (2)	0.15017 (18)	0.0494 (7)	
C44	0.3379 (2)	0.7675 (2)	0.08353 (17)	0.0448 (6)	
C40	0.4155 (2)	0.7680 (2)	0.25316 (18)	0.0497 (7)	
H40	0.4858	0.7804	0.2467	0.060*	
O10	0.68645 (17)	1.0687 (2)	0.25039 (17)	0.0800 (7)	
C39	0.4018 (2)	0.7572 (2)	0.33852 (17)	0.0478 (6)	
H39	0.4626	0.7614	0.3881	0.057*	
C29	−0.0186 (2)	0.3911 (2)	0.34652 (16)	0.0463 (6)	
H29	−0.0198	0.3304	0.3241	0.056*	
C45	0.2510 (3)	0.6889 (2)	0.00359 (18)	0.0566 (7)	
H45	0.1853	0.6356	0.0093	0.068*	
C27	0.2871 (3)	0.4091 (3)	0.3154 (3)	0.0810 (11)	
H27A	0.3425	0.4753	0.3115	0.121*	
H27B	0.2674	0.3564	0.2624	0.121*	
H27C	0.3210	0.3945	0.3721	0.121*	
C46	0.2609 (3)	0.6887 (3)	−0.0840 (2)	0.0695 (9)	
H46	0.2018	0.6357	−0.1360	0.083*	
C36	0.1841 (5)	1.0009 (3)	0.5278 (4)	0.1106 (17)	
H36A	0.2514	1.0233	0.5836	0.166*	
H36B	0.1417	1.0355	0.5371	0.166*	
H36C	0.2093	1.0174	0.4761	0.166*	
C35	0.1068 (3)	0.8872 (2)	0.5069 (3)	0.0742 (10)	
H35A	0.0828	0.8698	0.5596	0.089*	
H35B	0.0369	0.8646	0.4522	0.089*	
C6	−0.2024 (2)	0.0933 (2)	1.01931 (18)	0.0485 (6)	
H6A	−0.2768	0.0794	1.0165	0.058*	
C4	0.0015 (3)	0.1616 (2)	1.10673 (19)	0.0530 (7)	
H4	0.0630	0.1938	1.1644	0.064*	
N2	−0.26434 (18)	0.01972 (18)	0.84744 (14)	0.0486 (5)	
C8	−0.0689 (2)	0.08916 (19)	0.94074 (16)	0.0406 (6)	
C13	0.0667 (2)	0.1405 (2)	0.77654 (16)	0.0435 (6)	
N5	0.1701 (2)	0.28881 (17)	0.50981 (15)	0.0489 (6)	
C2	0.1521 (2)	0.1676 (2)	1.04685 (18)	0.0517 (7)	
N1	−0.08666 (17)	0.05155 (17)	0.84652 (14)	0.0442 (5)	
O3	−0.24105 (17)	−0.0311 (2)	0.70583 (13)	0.0698 (7)	
C23	0.4272 (2)	0.5237 (2)	0.6411 (2)	0.0602 (8)	
H23	0.4493	0.5274	0.5889	0.072*	
C7	−0.1820 (2)	0.06830 (19)	0.93793 (17)	0.0409 (6)	
C19	0.3071 (2)	0.4216 (2)	0.72559 (18)	0.0483 (6)	
C20	0.3596 (3)	0.5141 (3)	0.7955 (2)	0.0615 (8)	
H20	0.3371	0.5124	0.8474	0.074*	
O2	0.17589 (17)	0.1522 (2)	0.97154 (14)	0.0765 (7)	
O4	0.2578 (2)	0.22670 (19)	0.44503 (18)	0.0806 (7)	
C10	−0.3664 (3)	−0.0870 (3)	0.6532 (2)	0.0675 (9)	
H10A	−0.4086	−0.1139	0.6945	0.081*	
H10B	−0.3860	−0.1448	0.6042	0.081*	
C17	0.1361 (2)	0.3196 (2)	0.77355 (18)	0.0493 (6)	
H17	0.1300	0.3789	0.7849	0.059*	
C18	0.0615 (2)	0.2311 (2)	0.79280 (17)	0.0464 (6)	
H18	0.0067	0.2322	0.8171	0.056*	
C16	0.2204 (2)	0.3224 (2)	0.73761 (16)	0.0458 (6)	
C26	0.1515 (3)	0.2183 (2)	0.4336 (2)	0.0660 (9)	
O5	0.0632 (2)	0.1582 (2)	0.36806 (18)	0.0934 (9)	
C24	0.3419 (2)	0.4284 (2)	0.64672 (19)	0.0492 (6)	
C3	0.0267 (2)	0.13857 (19)	1.02678 (17)	0.0431 (6)	
C9	−0.2031 (2)	0.0113 (2)	0.79755 (17)	0.0475 (6)	
C14	0.1498 (2)	0.1413 (2)	0.73955 (19)	0.0513 (7)	
H14	0.1548	0.0815	0.7278	0.062*	
C11	−0.3998 (3)	−0.0156 (3)	0.6118 (3)	0.0865 (11)	
H11A	−0.3826	0.0401	0.6609	0.130*	
H11B	−0.4822	−0.0521	0.5752	0.130*	
H11C	−0.3563	0.0119	0.5723	0.130*	
C12	−0.0084 (2)	0.0423 (2)	0.80109 (18)	0.0468 (6)	
H12A	−0.0559	−0.0134	0.7441	0.056*	
H12B	0.0429	0.0233	0.8426	0.056*	
C25	0.2861 (2)	0.3394 (2)	0.56306 (19)	0.0495 (6)	
C5	−0.1081 (3)	0.1391 (2)	1.10392 (19)	0.0549 (7)	
H5	−0.1195	0.1547	1.1591	0.066*	
N6	0.3451 (2)	0.3062 (2)	0.5304 (2)	0.0703 (7)	
C15	0.2251 (2)	0.2304 (2)	0.7201 (2)	0.0537 (7)	
H15	0.2792	0.2291	0.6952	0.064*	
O1	0.2246 (2)	0.1998 (3)	1.12383 (17)	0.1087 (11)	
C21	0.4437 (3)	0.6082 (3)	0.7900 (3)	0.0750 (10)	
H21	0.4763	0.6679	0.8375	0.090*	
C1	0.2946 (3)	0.1770 (3)	0.9835 (3)	0.0774 (10)	
H1A	0.3383	0.2472	0.9805	0.116*	
H1B	0.2935	0.1310	0.9346	0.116*	
H1C	0.3311	0.1693	1.0434	0.116*	
C22	0.4786 (3)	0.6127 (3)	0.7140 (3)	0.0754 (10)	
H22	0.5364	0.6751	0.7111	0.091*	
O12	0.7580 (4)	0.5278 (3)	0.8989 (2)	0.1270 (12)	
O11	0.9139 (5)	0.5926 (4)	1.0263 (3)	0.177 (2)	
C55	0.8719 (6)	0.5628 (3)	0.9449 (3)	0.1081 (17)	
C56	0.9303 (4)	0.5469 (4)	0.8823 (4)	0.1221 (18)	
H56A	0.9792	0.6121	0.8723	0.183*	
H56B	0.8722	0.5002	0.8233	0.183*	
H56C	0.9777	0.5180	0.9106	0.183*	
C53	0.5742 (6)	0.4463 (7)	0.9154 (5)	0.155 (3)	
H53A	0.5438	0.4311	0.8481	0.233*	
H53B	0.5218	0.4571	0.9398	0.233*	
H53C	0.5814	0.3892	0.9340	0.233*	
C54	0.6848 (8)	0.5360 (6)	0.9508 (5)	0.173 (3)	
H54A	0.7225	0.5442	1.0170	0.207*	
H54B	0.6761	0.5963	0.9440	0.207*	
H5A	0.109 (3)	0.300 (2)	0.523 (2)	0.056 (8)*	
H7	0.428 (3)	1.002 (3)	0.187 (2)	0.077 (11)*	

### Atomic displacement parameters (Å^2^)

**Table:**

	*U*^11^	*U*^22^	*U*^33^	*U*^12^	*U*^13^	*U*^23^
N4	0.0429 (11)	0.0436 (12)	0.0375 (10)	0.0196 (10)	0.0170 (9)	0.0104 (9)
C32	0.0370 (12)	0.0442 (14)	0.0270 (10)	0.0173 (11)	0.0119 (9)	0.0113 (9)
C33	0.0349 (12)	0.0399 (13)	0.0249 (10)	0.0112 (10)	0.0105 (9)	0.0079 (9)
N3	0.0388 (10)	0.0372 (11)	0.0351 (10)	0.0133 (9)	0.0161 (8)	0.0105 (8)
O8	0.0601 (11)	0.0383 (10)	0.0667 (12)	0.0179 (9)	0.0269 (10)	0.0105 (9)
C28	0.0426 (13)	0.0478 (14)	0.0285 (11)	0.0216 (11)	0.0146 (10)	0.0106 (10)
C31	0.0325 (12)	0.0511 (16)	0.0357 (11)	0.0123 (11)	0.0112 (9)	0.0112 (11)
N7	0.0376 (12)	0.0498 (13)	0.0492 (12)	0.0155 (10)	0.0188 (10)	0.0125 (10)
O7	0.0651 (12)	0.0872 (16)	0.0610 (12)	0.0477 (12)	0.0256 (10)	0.0071 (11)
C42	0.0390 (13)	0.0466 (14)	0.0389 (12)	0.0161 (11)	0.0103 (10)	0.0139 (11)
C37	0.0346 (12)	0.0436 (14)	0.0382 (12)	0.0106 (11)	0.0120 (10)	0.0096 (10)
C43	0.0335 (12)	0.0492 (15)	0.0442 (13)	0.0156 (11)	0.0147 (10)	0.0158 (11)
C48	0.0737 (19)	0.083 (2)	0.0556 (16)	0.0522 (18)	0.0419 (15)	0.0360 (16)
N8	0.0481 (14)	0.094 (2)	0.0837 (18)	0.0316 (15)	0.0321 (13)	0.0201 (16)
C38	0.0341 (12)	0.0331 (12)	0.0367 (11)	0.0103 (10)	0.0116 (9)	0.0081 (9)
C51	0.0402 (14)	0.0634 (19)	0.0542 (16)	0.0102 (14)	0.0198 (12)	0.0218 (15)
C30	0.0388 (13)	0.0422 (14)	0.0365 (12)	0.0087 (11)	0.0094 (10)	0.0056 (10)
C47	0.104 (2)	0.085 (2)	0.0452 (16)	0.064 (2)	0.0411 (17)	0.0250 (16)
C41	0.0470 (14)	0.0376 (13)	0.0414 (12)	0.0184 (11)	0.0192 (11)	0.0136 (10)
O9	0.0623 (14)	0.0657 (15)	0.0689 (14)	0.0020 (11)	0.0222 (11)	−0.0045 (12)
C34	0.0468 (14)	0.0383 (13)	0.0331 (11)	0.0172 (11)	0.0145 (10)	0.0093 (10)
O6	0.0507 (12)	0.0819 (16)	0.0742 (14)	0.0383 (12)	0.0080 (11)	0.0090 (12)
C49	0.0532 (15)	0.0574 (16)	0.0476 (14)	0.0356 (13)	0.0277 (12)	0.0212 (12)
C52	0.0474 (15)	0.0520 (15)	0.0438 (13)	0.0271 (13)	0.0179 (12)	0.0180 (12)
C50	0.0459 (14)	0.0658 (18)	0.0513 (14)	0.0302 (13)	0.0284 (12)	0.0306 (13)
C44	0.0561 (15)	0.0480 (15)	0.0413 (13)	0.0305 (13)	0.0230 (11)	0.0172 (11)
C40	0.0442 (14)	0.0674 (18)	0.0489 (14)	0.0315 (13)	0.0232 (12)	0.0240 (13)
O10	0.0391 (11)	0.0911 (17)	0.0845 (16)	0.0140 (11)	0.0207 (11)	0.0165 (13)
C39	0.0394 (13)	0.0599 (17)	0.0413 (13)	0.0225 (12)	0.0133 (10)	0.0167 (12)
C29	0.0507 (14)	0.0424 (14)	0.0356 (12)	0.0166 (12)	0.0142 (11)	0.0041 (10)
C45	0.0722 (19)	0.0518 (17)	0.0434 (14)	0.0287 (15)	0.0201 (13)	0.0149 (12)
C27	0.077 (2)	0.112 (3)	0.083 (2)	0.064 (2)	0.0418 (19)	0.019 (2)
C46	0.102 (3)	0.065 (2)	0.0442 (15)	0.0459 (19)	0.0233 (15)	0.0082 (14)
C36	0.149 (4)	0.055 (2)	0.148 (4)	0.045 (2)	0.091 (4)	0.024 (2)
C35	0.090 (2)	0.0496 (18)	0.095 (2)	0.0353 (17)	0.050 (2)	0.0159 (17)
C6	0.0527 (15)	0.0542 (16)	0.0526 (15)	0.0314 (13)	0.0281 (12)	0.0185 (12)
C4	0.0603 (17)	0.0525 (16)	0.0419 (13)	0.0269 (14)	0.0165 (12)	0.0059 (12)
N2	0.0444 (12)	0.0584 (14)	0.0462 (12)	0.0263 (11)	0.0188 (9)	0.0145 (10)
C8	0.0462 (13)	0.0383 (13)	0.0439 (13)	0.0229 (11)	0.0213 (11)	0.0130 (10)
C13	0.0476 (14)	0.0560 (16)	0.0361 (12)	0.0314 (12)	0.0168 (10)	0.0151 (11)
N5	0.0532 (13)	0.0457 (13)	0.0501 (12)	0.0236 (11)	0.0251 (11)	0.0060 (10)
C2	0.0480 (15)	0.0460 (15)	0.0494 (15)	0.0205 (13)	0.0099 (12)	0.0015 (12)
N1	0.0410 (11)	0.0545 (13)	0.0408 (11)	0.0247 (10)	0.0177 (9)	0.0129 (9)
O3	0.0498 (11)	0.1100 (18)	0.0404 (10)	0.0368 (12)	0.0126 (8)	0.0065 (10)
C23	0.0451 (15)	0.0601 (18)	0.0777 (19)	0.0260 (14)	0.0231 (14)	0.0257 (16)
C7	0.0454 (13)	0.0412 (13)	0.0443 (13)	0.0249 (11)	0.0191 (11)	0.0187 (11)
C19	0.0442 (14)	0.0562 (16)	0.0476 (14)	0.0297 (13)	0.0115 (11)	0.0160 (12)
C20	0.0601 (17)	0.066 (2)	0.0509 (16)	0.0313 (16)	0.0114 (13)	0.0073 (14)
O2	0.0433 (11)	0.124 (2)	0.0536 (11)	0.0380 (12)	0.0153 (9)	0.0127 (12)
O4	0.0998 (18)	0.0762 (16)	0.0925 (17)	0.0526 (14)	0.0595 (15)	0.0127 (13)
C10	0.0561 (17)	0.077 (2)	0.0543 (16)	0.0256 (17)	0.0137 (14)	0.0107 (15)
C17	0.0638 (17)	0.0565 (16)	0.0459 (14)	0.0404 (14)	0.0254 (12)	0.0171 (12)
C18	0.0535 (15)	0.0652 (17)	0.0416 (13)	0.0415 (14)	0.0238 (11)	0.0201 (12)
C16	0.0490 (14)	0.0590 (16)	0.0368 (12)	0.0329 (13)	0.0142 (11)	0.0152 (11)
C26	0.085 (2)	0.0547 (18)	0.0666 (19)	0.0336 (17)	0.0434 (18)	0.0087 (16)
O5	0.1028 (19)	0.0788 (17)	0.0718 (15)	0.0287 (15)	0.0355 (14)	−0.0206 (13)
C24	0.0394 (13)	0.0524 (16)	0.0566 (15)	0.0226 (12)	0.0185 (11)	0.0143 (13)
C3	0.0470 (14)	0.0386 (13)	0.0451 (13)	0.0229 (11)	0.0157 (11)	0.0105 (11)
C9	0.0438 (14)	0.0573 (16)	0.0435 (13)	0.0254 (13)	0.0177 (11)	0.0130 (12)
C14	0.0632 (17)	0.0549 (16)	0.0604 (16)	0.0407 (14)	0.0342 (14)	0.0215 (13)
C11	0.065 (2)	0.082 (3)	0.092 (3)	0.0233 (19)	0.0231 (19)	0.026 (2)
C12	0.0454 (14)	0.0538 (16)	0.0454 (13)	0.0269 (12)	0.0200 (11)	0.0071 (12)
C25	0.0497 (15)	0.0544 (16)	0.0561 (15)	0.0286 (13)	0.0284 (13)	0.0212 (13)
C5	0.0701 (18)	0.0612 (18)	0.0462 (14)	0.0372 (15)	0.0302 (13)	0.0154 (13)
N6	0.0742 (17)	0.0773 (18)	0.0840 (18)	0.0489 (15)	0.0430 (15)	0.0210 (15)
C15	0.0577 (16)	0.0594 (17)	0.0657 (17)	0.0365 (14)	0.0371 (14)	0.0228 (14)
O1	0.0606 (15)	0.167 (3)	0.0615 (15)	0.0483 (17)	0.0000 (12)	−0.0188 (16)
C21	0.0578 (19)	0.056 (2)	0.083 (2)	0.0224 (16)	0.0038 (17)	−0.0044 (17)
C1	0.0463 (17)	0.103 (3)	0.080 (2)	0.0350 (18)	0.0241 (15)	0.018 (2)
C22	0.0520 (18)	0.055 (2)	0.102 (3)	0.0182 (15)	0.0180 (18)	0.0210 (19)
O12	0.140 (3)	0.168 (4)	0.0779 (19)	0.083 (3)	0.036 (2)	0.029 (2)
O11	0.254 (5)	0.171 (4)	0.086 (2)	0.140 (4)	−0.012 (3)	−0.009 (2)
C55	0.154 (5)	0.068 (3)	0.065 (2)	0.047 (3)	0.002 (3)	0.015 (2)
C56	0.106 (4)	0.123 (4)	0.113 (4)	0.043 (3)	0.021 (3)	0.053 (3)
C53	0.157 (6)	0.219 (8)	0.154 (5)	0.149 (6)	0.054 (5)	0.034 (5)
C54	0.221 (8)	0.181 (7)	0.127 (5)	0.102 (7)	0.081 (6)	0.015 (5)

### Geometric parameters (Å, º)

**Table:**

N4—C34	1.310 (3)	N2—C7	1.389 (3)
N4—C32	1.400 (3)	C8—C3	1.408 (4)
C32—C31	1.404 (3)	C8—N1	1.412 (3)
C32—C33	1.412 (3)	C8—C7	1.420 (3)
C33—C28	1.404 (4)	C13—C18	1.388 (4)
C33—N3	1.413 (3)	C13—C14	1.400 (3)
N3—C34	1.368 (3)	C13—C12	1.524 (4)
N3—C37	1.458 (3)	N5—C25	1.357 (4)
O8—C34	1.345 (3)	N5—C26	1.380 (4)
O8—C35	1.456 (4)	N5—H5A	1.00 (3)
C28—C29	1.409 (3)	C2—O1	1.193 (3)
C28—C52	1.505 (4)	C2—O2	1.313 (3)
C31—C30	1.373 (4)	C2—C3	1.495 (4)
C31—H31	0.9300	N1—C9	1.359 (3)
N7—C50	1.367 (4)	N1—C12	1.476 (3)
N7—C51	1.380 (4)	O3—C9	1.335 (3)
N7—H7	0.90 (4)	O3—C10	1.450 (4)
O7—C52	1.337 (3)	C23—C22	1.399 (5)
O7—C27	1.456 (4)	C23—C24	1.411 (4)
C42—C41	1.393 (4)	C23—H23	0.9300
C42—C43	1.398 (3)	C19—C20	1.407 (4)
C42—H42	0.9300	C19—C24	1.416 (4)
C37—C38	1.526 (3)	C19—C16	1.496 (4)
C37—H37A	0.9700	C20—C21	1.392 (5)
C37—H37B	0.9700	C20—H20	0.9300
C43—C38	1.376 (4)	O2—C1	1.445 (4)
C43—H43	0.9300	O4—C26	1.359 (4)
C48—C47	1.379 (5)	O4—N6	1.454 (4)
C48—C49	1.402 (4)	C10—C11	1.465 (6)
C48—H48	0.9300	C10—H10A	0.9700
N8—C50	1.288 (4)	C10—H10B	0.9700
N8—O10	1.454 (4)	C17—C18	1.383 (4)
C38—C39	1.388 (3)	C17—C16	1.396 (4)
C51—O9	1.219 (4)	C17—H17	0.9300
C51—O10	1.352 (4)	C18—H18	0.9300
C30—C29	1.403 (4)	C16—C15	1.407 (4)
C30—H30	0.9300	C26—O5	1.206 (4)
C47—C46	1.388 (5)	C24—C25	1.490 (4)
C47—H47	0.9300	C14—C15	1.394 (4)
C41—C40	1.384 (4)	C14—H14	0.9300
C41—C44	1.504 (3)	C11—H11A	0.9600
O6—C52	1.201 (3)	C11—H11B	0.9600
C49—C44	1.407 (4)	C11—H11C	0.9600
C49—C50	1.487 (4)	C12—H12A	0.9700
C44—C45	1.400 (4)	C12—H12B	0.9700
C40—C39	1.389 (4)	C25—N6	1.298 (4)
C40—H40	0.9300	C5—H5	0.9300
C39—H39	0.9300	C15—H15	0.9300
C29—H29	0.9300	C21—C22	1.373 (5)
C45—C46	1.388 (4)	C21—H21	0.9300
C45—H45	0.9300	C1—H1A	0.9600
C27—H27A	0.9600	C1—H1B	0.9600
C27—H27B	0.9600	C1—H1C	0.9600
C27—H27C	0.9600	C22—H22	0.9300
C46—H46	0.9300	O12—C55	1.336 (6)
C36—C35	1.485 (5)	O12—C54	1.478 (8)
C36—H36A	0.9600	O11—C55	1.164 (5)
C36—H36B	0.9600	C55—C56	1.474 (8)
C36—H36C	0.9600	C56—H56A	0.9600
C35—H35A	0.9700	C56—H56B	0.9600
C35—H35B	0.9700	C56—H56C	0.9600
C6—C5	1.387 (4)	C53—C54	1.403 (9)
C6—C7	1.406 (4)	C53—H53A	0.9600
C6—H6A	0.9300	C53—H53B	0.9600
C4—C5	1.367 (4)	C53—H53C	0.9600
C4—C3	1.416 (4)	C54—H54A	0.9700
C4—H4	0.9300	C54—H54B	0.9700
N2—C9	1.318 (3)		
			
C34—N4—C32	104.0 (2)	C18—C13—C12	123.8 (2)
N4—C32—C31	129.7 (2)	C14—C13—C12	118.3 (2)
N4—C32—C33	110.76 (19)	C25—N5—C26	108.1 (3)
C31—C32—C33	119.5 (2)	C25—N5—H5A	126.5 (16)
C28—C33—C32	121.6 (2)	C26—N5—H5A	125.4 (17)
C28—C33—N3	134.1 (2)	O1—C2—O2	122.1 (3)
C32—C33—N3	104.3 (2)	O1—C2—C3	124.0 (3)
C34—N3—C33	105.87 (19)	O2—C2—C3	113.9 (2)
C34—N3—C37	123.7 (2)	C9—N1—C8	106.07 (19)
C33—N3—C37	129.9 (2)	C9—N1—C12	121.2 (2)
C34—O8—C35	116.7 (2)	C8—N1—C12	132.5 (2)
C33—C28—C29	117.4 (2)	C9—O3—C10	118.6 (2)
C33—C28—C52	124.0 (2)	C22—C23—C24	120.0 (3)
C29—C28—C52	118.2 (2)	C22—C23—H23	120.0
C30—C31—C32	119.2 (2)	C24—C23—H23	120.0
C30—C31—H31	120.4	N2—C7—C6	127.0 (2)
C32—C31—H31	120.4	N2—C7—C8	111.1 (2)
C50—N7—C51	108.3 (2)	C6—C7—C8	121.9 (2)
C50—N7—H7	129 (2)	C20—C19—C24	116.7 (3)
C51—N7—H7	122 (2)	C20—C19—C16	119.8 (2)
C52—O7—C27	115.1 (2)	C24—C19—C16	123.4 (2)
C41—C42—C43	120.5 (2)	C21—C20—C19	122.6 (3)
C41—C42—H42	119.7	C21—C20—H20	118.7
C43—C42—H42	119.7	C19—C20—H20	118.7
N3—C37—C38	113.43 (18)	C2—O2—C1	118.2 (2)
N3—C37—H37A	108.9	C26—O4—N6	109.2 (2)
C38—C37—H37A	108.9	O3—C10—C11	108.5 (3)
N3—C37—H37B	108.9	O3—C10—H10A	110.0
C38—C37—H37B	108.9	C11—C10—H10A	110.0
H37A—C37—H37B	107.7	O3—C10—H10B	110.0
C38—C43—C42	121.1 (2)	C11—C10—H10B	110.0
C38—C43—H43	119.5	H10A—C10—H10B	108.4
C42—C43—H43	119.5	C18—C17—C16	121.8 (3)
C47—C48—C49	121.1 (3)	C18—C17—H17	119.1
C47—C48—H48	119.4	C16—C17—H17	119.1
C49—C48—H48	119.4	C17—C18—C13	121.2 (2)
C50—N8—O10	104.1 (3)	C17—C18—H18	119.4
C43—C38—C39	118.3 (2)	C13—C18—H18	119.4
C43—C38—C37	121.8 (2)	C17—C16—C15	117.1 (2)
C39—C38—C37	119.8 (2)	C17—C16—C19	120.7 (3)
O9—C51—O10	124.3 (3)	C15—C16—C19	122.0 (2)
O9—C51—N7	130.4 (3)	O5—C26—O4	123.2 (3)
O10—C51—N7	105.3 (3)	O5—C26—N5	131.0 (3)
C31—C30—C29	121.6 (2)	O4—C26—N5	105.8 (3)
C31—C30—H30	119.2	C23—C24—C19	120.7 (3)
C29—C30—H30	119.2	C23—C24—C25	116.2 (2)
C48—C47—C46	119.0 (3)	C19—C24—C25	122.9 (2)
C48—C47—H47	120.5	C8—C3—C4	116.5 (2)
C46—C47—H47	120.5	C8—C3—C2	129.0 (2)
C40—C41—C42	118.1 (2)	C4—C3—C2	114.5 (2)
C40—C41—C44	121.5 (2)	N2—C9—O3	128.1 (2)
C42—C41—C44	120.3 (2)	N2—C9—N1	115.2 (2)
N4—C34—O8	127.7 (3)	O3—C9—N1	116.7 (2)
N4—C34—N3	115.0 (2)	C15—C14—C13	120.9 (3)
O8—C34—N3	117.2 (2)	C15—C14—H14	119.5
C48—C49—C44	120.2 (3)	C13—C14—H14	119.5
C48—C49—C50	118.1 (2)	C10—C11—H11A	109.5
C44—C49—C50	121.4 (2)	C10—C11—H11B	109.5
O6—C52—O7	123.9 (3)	H11A—C11—H11B	109.5
O6—C52—C28	123.6 (2)	C10—C11—H11C	109.5
O7—C52—C28	112.5 (2)	H11A—C11—H11C	109.5
N8—C50—N7	112.5 (3)	H11B—C11—H11C	109.5
N8—C50—C49	123.6 (3)	N1—C12—C13	114.3 (2)
N7—C50—C49	123.8 (2)	N1—C12—H12A	108.7
C45—C44—C49	117.7 (2)	C13—C12—H12A	108.7
C45—C44—C41	119.2 (2)	N1—C12—H12B	108.7
C49—C44—C41	123.1 (2)	C13—C12—H12B	108.7
C41—C40—C39	121.0 (2)	H12A—C12—H12B	107.6
C41—C40—H40	119.5	N6—C25—N5	112.9 (3)
C39—C40—H40	119.5	N6—C25—C24	123.1 (3)
C51—O10—N8	109.8 (2)	N5—C25—C24	123.9 (2)
C38—C39—C40	120.9 (2)	C4—C5—C6	120.4 (2)
C38—C39—H39	119.5	C4—C5—H5	119.8
C40—C39—H39	119.5	C6—C5—H5	119.8
C30—C29—C28	120.6 (2)	C25—N6—O4	104.0 (2)
C30—C29—H29	119.7	C14—C15—C16	121.0 (2)
C28—C29—H29	119.7	C14—C15—H15	119.5
C46—C45—C44	121.4 (3)	C16—C15—H15	119.5
C46—C45—H45	119.3	C22—C21—C20	119.8 (3)
C44—C45—H45	119.3	C22—C21—H21	120.1
O7—C27—H27A	109.5	C20—C21—H21	120.1
O7—C27—H27B	109.5	O2—C1—H1A	109.5
H27A—C27—H27B	109.5	O2—C1—H1B	109.5
O7—C27—H27C	109.5	H1A—C1—H1B	109.5
H27A—C27—H27C	109.5	O2—C1—H1C	109.5
H27B—C27—H27C	109.5	H1A—C1—H1C	109.5
C45—C46—C47	120.5 (3)	H1B—C1—H1C	109.5
C45—C46—H46	119.7	C21—C22—C23	120.2 (3)
C47—C46—H46	119.7	C21—C22—H22	119.9
C35—C36—H36A	109.5	C23—C22—H22	119.9
C35—C36—H36B	109.5	C55—O12—C54	119.6 (5)
H36A—C36—H36B	109.5	O11—C55—O12	121.7 (6)
C35—C36—H36C	109.5	O11—C55—C56	125.8 (6)
H36A—C36—H36C	109.5	O12—C55—C56	112.1 (4)
H36B—C36—H36C	109.5	C55—C56—H56A	109.5
O8—C35—C36	109.0 (3)	C55—C56—H56B	109.5
O8—C35—H35A	109.9	H56A—C56—H56B	109.5
C36—C35—H35A	109.9	C55—C56—H56C	109.5
O8—C35—H35B	109.9	H56A—C56—H56C	109.5
C36—C35—H35B	109.9	H56B—C56—H56C	109.5
H35A—C35—H35B	108.3	C54—C53—H53A	109.5
C5—C6—C7	118.0 (2)	C54—C53—H53B	109.5
C5—C6—H6A	121.0	H53A—C53—H53B	109.5
C7—C6—H6A	121.0	C54—C53—H53C	109.5
C5—C4—C3	123.7 (3)	H53A—C53—H53C	109.5
C5—C4—H4	118.1	H53B—C53—H53C	109.5
C3—C4—H4	118.1	C53—C54—O12	109.4 (6)
C9—N2—C7	103.8 (2)	C53—C54—H54A	109.8
C3—C8—N1	136.7 (2)	O12—C54—H54A	109.8
C3—C8—C7	119.5 (2)	C53—C54—H54B	109.8
N1—C8—C7	103.9 (2)	O12—C54—H54B	109.8
C18—C13—C14	117.9 (2)	H54A—C54—H54B	108.2
			
C34—N4—C32—C31	180.0 (2)	C3—C8—N1—C12	5.7 (5)
C34—N4—C32—C33	1.3 (2)	C7—C8—N1—C12	−175.2 (3)
N4—C32—C33—C28	−179.2 (2)	C9—N2—C7—C6	−178.2 (3)
C31—C32—C33—C28	2.0 (3)	C9—N2—C7—C8	0.2 (3)
N4—C32—C33—N3	−0.6 (2)	C5—C6—C7—N2	178.0 (3)
C31—C32—C33—N3	−179.5 (2)	C5—C6—C7—C8	−0.2 (4)
C28—C33—N3—C34	178.0 (2)	C3—C8—C7—N2	179.9 (2)
C32—C33—N3—C34	−0.3 (2)	N1—C8—C7—N2	0.6 (3)
C28—C33—N3—C37	6.6 (4)	C3—C8—C7—C6	−1.6 (4)
C32—C33—N3—C37	−171.7 (2)	N1—C8—C7—C6	179.1 (2)
C32—C33—C28—C29	0.2 (3)	C24—C19—C20—C21	−1.5 (5)
N3—C33—C28—C29	−177.9 (2)	C16—C19—C20—C21	177.8 (3)
C32—C33—C28—C52	−172.4 (2)	O1—C2—O2—C1	−1.0 (5)
N3—C33—C28—C52	9.5 (4)	C3—C2—O2—C1	−179.5 (3)
N4—C32—C31—C30	177.6 (2)	C9—O3—C10—C11	93.9 (3)
C33—C32—C31—C30	−3.8 (3)	C16—C17—C18—C13	−0.4 (4)
C34—N3—C37—C38	−99.7 (3)	C14—C13—C18—C17	−0.3 (4)
C33—N3—C37—C38	70.3 (3)	C12—C13—C18—C17	176.6 (2)
C41—C42—C43—C38	−0.5 (4)	C18—C17—C16—C15	1.1 (4)
C42—C43—C38—C39	−1.0 (4)	C18—C17—C16—C19	−174.4 (2)
C42—C43—C38—C37	176.4 (2)	C20—C19—C16—C17	41.4 (4)
N3—C37—C38—C43	23.8 (3)	C24—C19—C16—C17	−139.3 (3)
N3—C37—C38—C39	−158.9 (2)	C20—C19—C16—C15	−133.8 (3)
C50—N7—C51—O9	177.4 (3)	C24—C19—C16—C15	45.4 (4)
C50—N7—C51—O10	0.1 (3)	N6—O4—C26—O5	179.7 (3)
C32—C31—C30—C29	3.6 (4)	N6—O4—C26—N5	−0.9 (3)
C49—C48—C47—C46	0.8 (5)	C25—N5—C26—O5	−178.9 (4)
C43—C42—C41—C40	1.3 (4)	C25—N5—C26—O4	1.8 (3)
C43—C42—C41—C44	177.9 (2)	C22—C23—C24—C19	0.2 (4)
C32—N4—C34—O8	175.5 (2)	C22—C23—C24—C25	175.9 (3)
C32—N4—C34—N3	−1.6 (3)	C20—C19—C24—C23	1.5 (4)
C35—O8—C34—N4	2.0 (4)	C16—C19—C24—C23	−177.8 (3)
C35—O8—C34—N3	179.0 (2)	C20—C19—C24—C25	−174.0 (3)
C33—N3—C34—N4	1.2 (3)	C16—C19—C24—C25	6.7 (4)
C37—N3—C34—N4	173.32 (19)	N1—C8—C3—C4	−179.2 (3)
C33—N3—C34—O8	−176.2 (2)	C7—C8—C3—C4	1.9 (4)
C37—N3—C34—O8	−4.1 (3)	N1—C8—C3—C2	−1.5 (5)
C47—C48—C49—C44	0.7 (5)	C7—C8—C3—C2	179.6 (3)
C47—C48—C49—C50	−173.5 (3)	C5—C4—C3—C8	−0.4 (4)
C27—O7—C52—O6	−4.3 (4)	C5—C4—C3—C2	−178.5 (3)
C27—O7—C52—C28	176.3 (2)	O1—C2—C3—C8	−172.0 (3)
C33—C28—C52—O6	30.0 (4)	O2—C2—C3—C8	6.5 (4)
C29—C28—C52—O6	−142.6 (3)	O1—C2—C3—C4	5.8 (5)
C33—C28—C52—O7	−150.6 (2)	O2—C2—C3—C4	−175.8 (3)
C29—C28—C52—O7	36.8 (3)	C7—N2—C9—O3	179.3 (3)
O10—N8—C50—N7	0.4 (3)	C7—N2—C9—N1	−1.1 (3)
O10—N8—C50—C49	176.6 (2)	C10—O3—C9—N2	−8.4 (5)
C51—N7—C50—N8	−0.3 (3)	C10—O3—C9—N1	172.0 (3)
C51—N7—C50—C49	−176.5 (2)	C8—N1—C9—N2	1.5 (3)
C48—C49—C50—N8	−58.7 (4)	C12—N1—C9—N2	176.4 (2)
C44—C49—C50—N8	127.2 (3)	C8—N1—C9—O3	−178.8 (2)
C48—C49—C50—N7	117.1 (3)	C12—N1—C9—O3	−3.9 (4)
C44—C49—C50—N7	−57.0 (4)	C18—C13—C14—C15	0.3 (4)
C48—C49—C44—C45	−1.7 (4)	C12—C13—C14—C15	−176.8 (2)
C50—C49—C44—C45	172.3 (3)	C9—N1—C12—C13	102.2 (3)
C48—C49—C44—C41	176.1 (3)	C8—N1—C12—C13	−84.5 (3)
C50—C49—C44—C41	−9.9 (4)	C18—C13—C12—N1	−1.5 (4)
C40—C41—C44—C45	126.8 (3)	C14—C13—C12—N1	175.4 (2)
C42—C41—C44—C45	−49.7 (4)	C26—N5—C25—N6	−2.1 (3)
C40—C41—C44—C49	−50.9 (4)	C26—N5—C25—C24	174.1 (3)
C42—C41—C44—C49	132.6 (3)	C23—C24—C25—N6	58.8 (4)
C42—C41—C40—C39	−0.6 (4)	C19—C24—C25—N6	−125.5 (3)
C44—C41—C40—C39	−177.2 (3)	C23—C24—C25—N5	−117.0 (3)
O9—C51—O10—N8	−177.4 (3)	C19—C24—C25—N5	58.7 (4)
N7—C51—O10—N8	0.1 (3)	C3—C4—C5—C6	−1.5 (5)
C50—N8—O10—C51	−0.3 (3)	C7—C6—C5—C4	1.8 (4)
C43—C38—C39—C40	1.7 (4)	N5—C25—N6—O4	1.4 (3)
C37—C38—C39—C40	−175.7 (3)	C24—C25—N6—O4	−174.8 (3)
C41—C40—C39—C38	−0.9 (4)	C26—O4—N6—C25	−0.3 (3)
C31—C30—C29—C28	−1.4 (4)	C13—C14—C15—C16	0.4 (4)
C33—C28—C29—C30	−0.5 (3)	C17—C16—C15—C14	−1.1 (4)
C52—C28—C29—C30	172.6 (2)	C19—C16—C15—C14	174.4 (3)
C49—C44—C45—C46	1.3 (5)	C19—C20—C21—C22	−0.2 (5)
C41—C44—C45—C46	−176.6 (3)	C20—C21—C22—C23	1.9 (5)
C44—C45—C46—C47	0.2 (5)	C24—C23—C22—C21	−1.9 (5)
C48—C47—C46—C45	−1.2 (5)	C54—O12—C55—O11	5.9 (8)
C34—O8—C35—C36	−174.6 (3)	C54—O12—C55—C56	179.2 (6)
C3—C8—N1—C9	179.8 (3)	C55—O12—C54—C53	−140.9 (6)
C7—C8—N1—C9	−1.2 (3)		

### Hydrogen-bond geometry (Å, º)

**Table:**

*D*—H···*A*	*D*—H	H···*A*	*D*···*A*	*D*—H···*A*
N5—H5*A*···N4^i^	1.01	1.85	2.862 (3)	176
N7—H7···N2^i^	0.90	2.01	2.905 (3)	173
C12—H12*A*···O5^ii^	0.97	2.58	3.421 (4)	145
C30—H30···O9^iii^	0.93	2.58	3.484 (3)	163

Symmetry codes: (i) −*x*, −*y*+1, −*z*+1; (ii) −*x*, −*y*, −*z*+1; (iii) *x*−1, *y*−1, *z*.
