# Supplementary figures and images for: Crystal structure of azilsartan methyl ester ethyl acetate hemisolvate
Source: Acta Crystallogr E Crystallogr Commun. 2015 Jan 3;71(Pt 2):o84–5. doi: 10.1107/S2056989014028023 (PMC4384622; doi:10.1107/S2056989014028023)

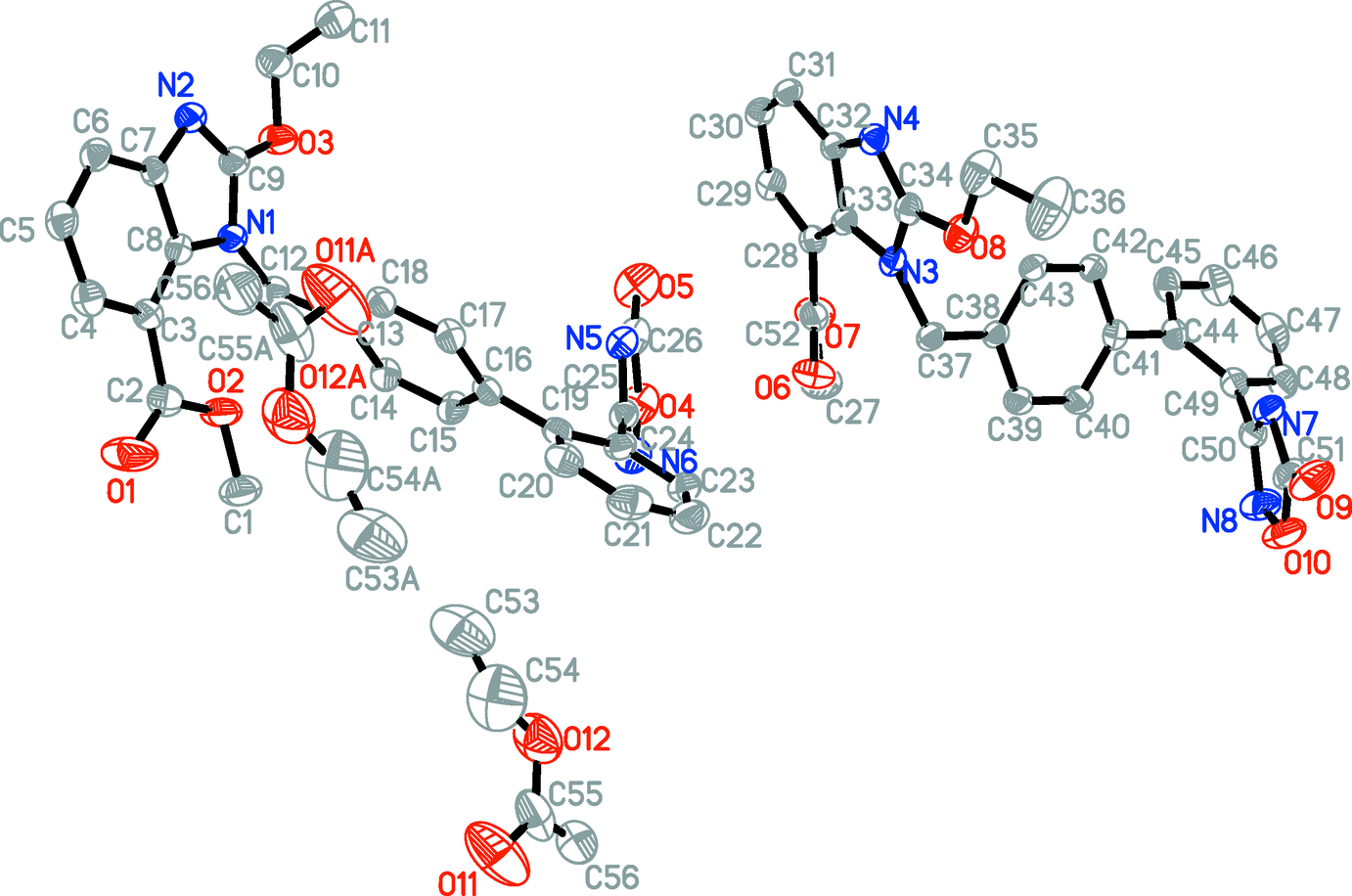

Supplement: Supplementary file 4 [file e-71-00o84-fig1.tif]

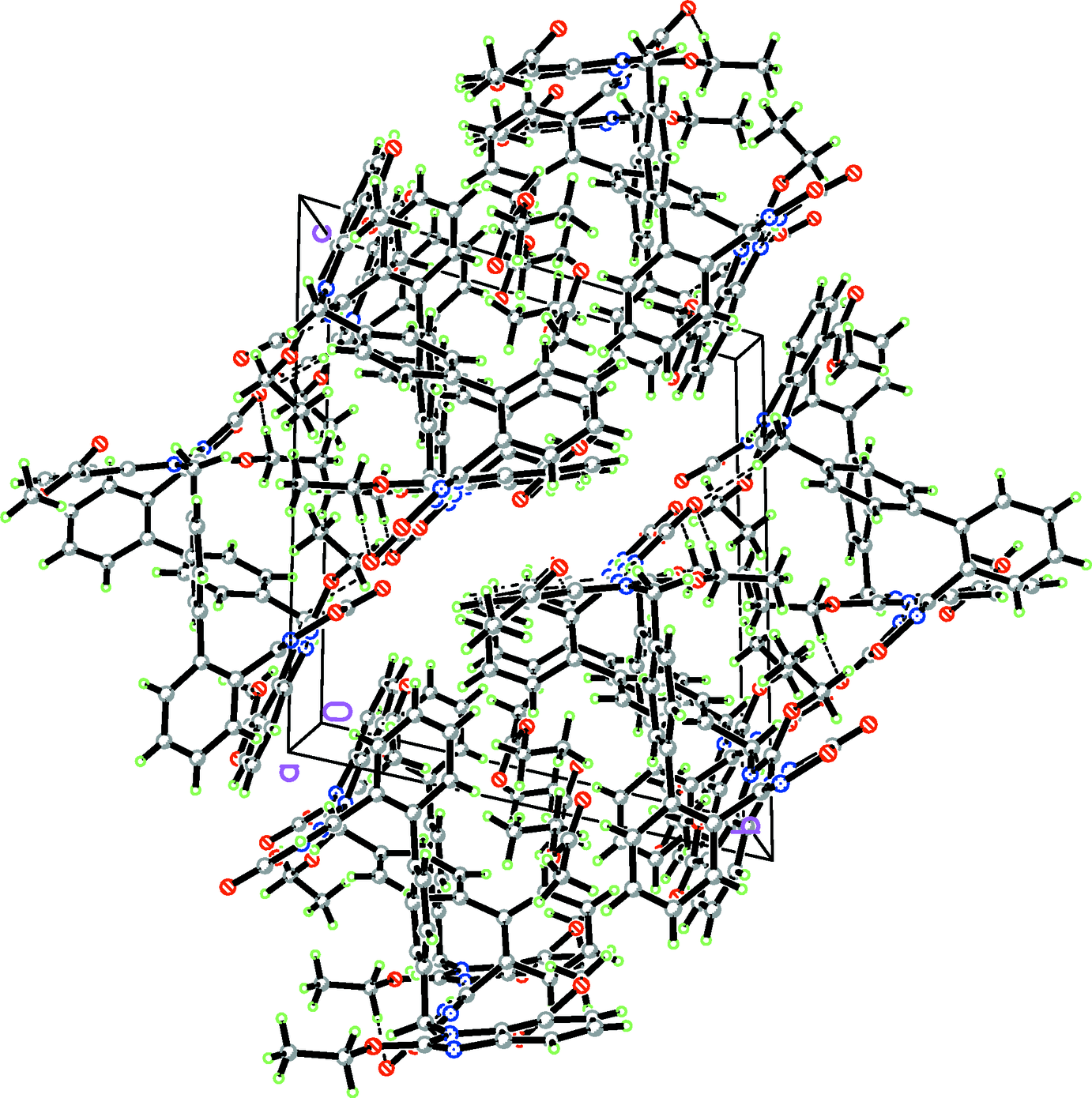

Supplement: Supplementary file 5 [file e-71-00o84-fig2.tif]
